# Therapy for metastatic melanoma: the past, present, and future

**DOI:** 10.1186/1741-7015-10-23

**Published:** 2012-03-02

**Authors:** Laura Finn, Svetomir N Markovic, Richard W Joseph

**Affiliations:** 1 Division of Hematology and Oncology, Mayo Clinic Florida, 4500 San Pablo Road, Jacksonville, FL 32224, USA; 2 Division of Hematology and Oncology, Mayo Clinic Rochester, Gonda Building 10 South, Rochester, MN 55905, USA

**Keywords:** Melanoma, Vemurafenib, Ipilimumab, BRAF, Therapy

## Abstract

Metastatic melanoma is the most aggressive form of skin cancer with a median overall survival of less than one year. Advancements in our understanding of how melanoma evades the immune system as well as the recognition that melanoma is a molecularly heterogeneous disease have led to major improvements in the treatment of patients with metastatic melanoma. In 2011, the US Food and Drug Administration (FDA) approved two novel therapies for advanced melanoma: a BRAF inhibitor, vemurafenib, and an immune stimulatory agent, ipilimumab. The success of these agents has injected excitement and hope into patients and clinicians and, while these therapies have their limitations, they will likely provide excellent building blocks for the next generation of therapies. In this review we will discuss the advantages and limitations of the two new approved agents, current clinical trials designed to overcome these limitations, and future clinical trials that we feel hold the most promise.

## Introduction

With approximately 13,000 annual deaths and a median overall survival (OS) of 8 to 18 months, metastatic melanoma is the most aggressive form of skin cancer [1]. Until 2011, only two FDA therapies for metastatic melanoma were approved, dacarbazine and high dose interleukin 2 (HD IL-2), both of which do not increase median OS [2-4]. Dacarbazine is limited by a low response rate (10% to 15%) and an overall survival of eight months [2]. HD IL-2 is limited by an even lower response rate (6% to 10%) and severe toxicity with only a minority of patients achieving a long-term, durable response [3,4].

Recognition of key molecular mutations that drive tumorigenesis in melanoma has led to the development of promising agents that selectively target and inhibit these mutations and, in turn, provide improved response rates with decreased toxicity. Secondarily, advancements in our understanding of tumor immunology and immune escape have led to the emergence of newer immunologic agents that are less toxic than HD IL-2 but still provide long-term benefits. While these breakthroughs are encouraging, several limitations remain. In the case of vemurafenib, the duration of response is relatively short. In the case with ipilimumab, the response rate is low. The purpose of this review is to summarize the recent advances in the treatment of metastatic melanoma, further describe the current limitations, and comment on promising future strategies to overcome these limitations.

### Recent advances

#### BRAF inhibitors

In 2002, it was discovered that cutaneous melanoma is a molecularly heterogeneous disease with approximately 40% to 60% harboring an activating mutation in the gene encoding for the serine/threonine kinase protein kinase B-raf (BRAF) with 90% of the mutations resulting in a substitution of valine for glutamate at amino acid 600 (V600E) [5-8]. Mutated BRAF leads to constitutive activation of the mitogen-activated protein kinase pathway (MAPK) that in turn increases cellular proliferation and drives oncogenic activity. Given the relatively high incidence of mutant BRAF as well as its oncogenic potential, investigators have long sought to selectively inhibit mutated BRAF. Earlier attempts to inhibit BRAF in patients with melanoma with sorafenib were largely unsuccessful secondary to the poor sensitivity of sorafenib to selectively target mutant BRAF that led to intolerable off-target side effects through inhibition of wild-type BRAF and other off-target effects [9-13]. Recently, highly selective BRAF inhibitors capable of silencing mutant BRAF (V600E) with little effect on wild-type BRAF have emerged (Table 1). In a phase 1 study, the first of these selective BRAF inhibitors, vemurafenib, demonstrated substantial tumor regression in 81% of patients with metastatic melanoma who had a BRAF (V600E) mutation and received the recommended phase 2 dose [13,14]. The follow up phase 2 (BRIM2) study of previously treated patients demonstrated a confirmed response rate (RR) of 53% with a 6.8 month median duration of response [15]. Finally, a phase 3 randomized control trial (BRIM3) of previously untreated patients compared vemurafenib to dacarbazine demonstrating improvements in RR (48% versus 5%), progression free survival (5.3 versus 1.6 months), percent of patients alive at six months (84% versus 64%) with a 75% reduction in risk of death [16]. A second BRAF inhibitor, GSK2118436, showed similar efficacy in a phase 1/2 study although OS data are not yet mature [17]. In addition, 10% to 30% of patients with a BRAF mutation have a non-V600E mutation with the most common non-V600E mutation being V600K which is present in 5% to 20% of melanoma patients with a BRAF mutation [7,18]. Both vemurafenib and GSK2118436 have shown activity in V600K mutant melanomas and while vemurafenib is not currently approved for patients with V600K mutations, further studies are examining its efficacy in non-V600E mutant patients [16,19]. Finally, both vemurafenib and GSK2118436 have been tested in patients with brain metastasis with apparent activity in the brain although the number of treated patients remains small at present [19,20]. In summary, both highly selective BRAF inhibitors, vemurafenib and GSK2118436, have demonstrated excellent clinical activity with a high response rate and low toxicity in patients with BRAF V600E mutations but, unfortunately, both therapies are limited by a relatively short duration that averages around six months. The most important toxicity related to BRAF inhibitors is accelerated growth of cutaneous squamous cell carcinomas (SCC) and keratoacanthomas through paradoxical activation of MAPK signaling occurring in approximately 20% of patients [21]. Fortunately, these SCC are easily removed and cured through local excision.

**Table 1:** Summary of BRAF inhibitor trials

Trial	Patients	RR%	PFS (months)	OS (years)	Number (Patients)	Summary
Phase 2 Ribas *et al. *[15]	Vemurafenib in previously treated metastatic melanoma	52	6.2	Not yet reached	132	Met primary end point of best overall survival target of 30% (95% CI: 43 to 61%)
Phase 3 Chapman *et al. *[16]	Vemurafenib versus dacarbazine in untreated metastatic melanoma	48 v 5	5.3 v 1.6	Not yet reached	675	Compared vemurafenib and dacarbazine with co-primary endpoints of overall survival and progression free survival. 84% vs 64% OS at 6 months (95% CI: 78 to 89)

OS, overall survival; PFS, progression free survival; RR, response rate.

#### Ipilimumab

Melanoma is characterized as one of the most immunogenic tumors due to the presence of tumor infiltrating lymphocytes in resected melanoma, occasional spontaneous regressions, and clinical responses to immune stimulation. The immunogenicity of melanoma has led investigators to study novel immune strategies to overcome tumor immune evasion. One mechanism by which T cells self-regulate their activation is through expression of cytotoxic T-lymphocyte-associated antigen 4 (CTLA-4). CTLA-4 functions as a negative co-stimulatory molecule for the T cell, and therapies that antagonize CTLA-4 remove the brakes from the T cell leading to a net effect of T cell hyper-responsiveness [22]. Ipilimumab is a fully human IgG1 monoclonal antibody that blocks CTLA-4, thereby increasing T-cell activity and promoting antitumor activity [23]. Two phase 3 randomized clinical trials have evaluated ipilimumab in metastatic melanoma [23,24]. In the first trial of patients with previously treated unresectable stage III or IV melanoma, ipilimumab demonstrated an improved overall survival versus glycoprotein 100 peptide vaccine (gp100) (10.1 versus 6.4 months) [24]. In the second phase 3 trial in previously untreated patients with metastatic melanoma, ipilimumab plus dacarbazine demonstrated improvement in OS versus single agent dacarbazine (11.2 versus 9.2 months) [23]. In both phase 3 studies, the response rate, complete response (CR) and partial response (PR) was only 10% to 15% and the disease control rate (CR, PR, and stable disease (SD)) was approximately 30%. In addition, the improvement in percent of patients alive at one and two years is consistently 10% better than the non-ipilimumab containing arms (Table 2). While the response rate and improvement in OS in ipilimumab is relatively modest, the toxicities of the therapy, including immune-related enterocolitis, hepatitis, and dermatitis, are highly manageable [24].

**Table 2:** Summary of Ipilimumab Trials

Trial	Patients	RR% (CR/PR)	PFS (months)	OS **(years**)	Number (patients)	Summary
Phase 3 Hodi *et al. *[24]	Ipilimumab plus gp100 versus Ipilimumab alone versus gp100 alone previously treated metastatic melanoma	5.7 versus 10.9 versus 1.5	2.76 versus 2.86 versus 2.76	10.0 versus 10.1 versus 6.4	676	Ipilimumab with significant improvement in OS versus gp-100.
Phase 3 Robert *et al. *[23]	Ipilimumab plus dacarbazine versus dacarbazine in untreated metastatic melanoma	15.2 versus 10.3	Not stated	11.2 versus 9.1	502	Ipilimumab plus dacarbazine significantly with significant improvement in OS over dacarbazine.
Phase 2 Hersh *et al. *[40]	Ipilimumab versus Ipilimumab plus dacarbazine in chemotherapy naïve patients with metastatic melanoma	5.3 versus 14.3	Not stated	11.4 versus 14.4	72	Ipilimumab plus dacarbazine improved RR and OS compared to single agent ipilimumab.

CR, complete response; OS, overall survival; PFS, progression free survival; PR, partial response; RR, response rate.

#### Future strategies

The recent success of both vemurafenib and ipilimumab instilled hope into physicians and patients with metastatic melanoma; however, the limitations of both therapies emphasize the importance of developing novel treatment strategies. One of the major challenges in overcoming the limitations of these novel therapies is both increasing the duration of response to BRAF inhibitors and improving the response rate of ipilimumab. We will discuss multiple strategies to address the limitations of vemurafenib below.

### Overcoming resistance to BRAF inhibitors using additional targeted therapies

**Figure 1:**
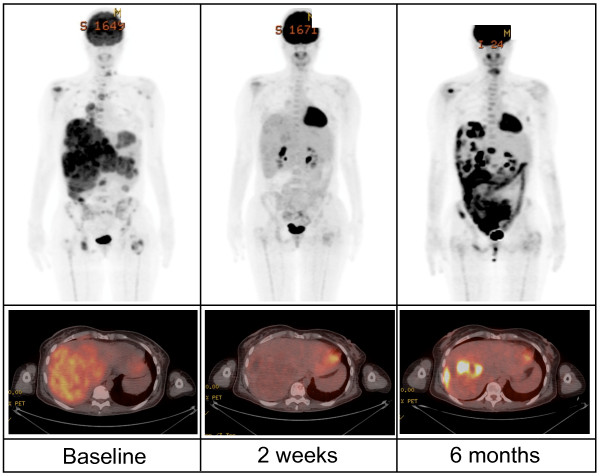
**Typical response for patients on BRAF inhibitors**. BRAF inhibitors can induce positron emission tomography (PET) responses in as little as two weeks but unfortunately most patients developed relapse and progressive disease at about six months. This patient was treated in the phase 1 study of PLX4032 (vermurafenib).

While selective BRAF inhibitors have provided a major breakthrough in the treatment of melanoma, resistance to therapy invariably develops with the median duration of benefit of approximately six months (Figure 1). Mutant BRAF drives melanoma tumor proliferation through activation of the mitogen activated kinase pathway (MAP) pathway, and resistance to BRAF inhibitors has been described in both MAP kinase-dependent and -independent pathways [25-27]. MAP kinase-dependent pathways of resistance include secondary NRAS mutations, elevated expression of COT kinase, CRAF activation, and acquired MEK1 mutation [25,28-31]. MAP kinase-independent pathways include upregulation of PDGFR, additional receptor tyrosine kinases activation including AXL, ERBB4, and IGF1R, activation of PI3K/AKT signaling, and loss of PTEN [25,29,31-35]. Strategies to overcoming these mechanisms of resistance are currently being developed. A recent phase 1/2 study combining an oral MEK 1/2 inhibitor GSK1120212 with BRAF inhibitor GSK2118436 was well tolerated [36], but it is too early to determine if the combination will increase the duration of response of a single agent BRAF inhibitor. A randomized phase 3 study set to begin in 2012 will hopefully address this question. Additional future targeted combinations will likely include BRAF inhibitors with PI3K, PDGFR, IGF1R, MEK, or ERBB4 inhibitors and will be expanded upon below.

### Improving upon ipilimumab

While ipilimumab is capable of inducing long-term responses in a minority of patients, the relatively low response rate (10% to 15%) and meager improvement in median survival (two months) limit its utility. A key area of improving the clinical benefit of ipilimumab is to increase the response rate either through improved patient selection or through combination with other therapies. At present, there is no reliable predictor of benefit for ipilimumab. One group recently reported that the presence of a BRAF mutation does not predict clinical benefit of ipilimumab [37]. Other groups have shown that ipilimumab increases the frequency of T cells with inducible co-stimulatory molecule (ICOS) [38] and that ICOS T cells are necessary for response to ipilimumab [39]. Whether or not baseline ICOS T cells predict benefit to ipilimumab remains to be determined. Finally, there is ample evidence to suggest that a higher dose of ipilimumab (10 mg/kg) results in an increased response than the approved dose (3 mg/kg) and a randomized phase 3 study is underway to address this question.

In addition to improved patient identification, many investigators are combining ipilimumab with other treatment modalities in order to increase the response rate. As mentioned above, the phase 3 study of dacarbazine plus ipilimumab did not yield a higher than expected response rate; however, this study was not designed to answer this question. In a randomized phase 2 study of ipilimumab versus ipilimumab and dacarbazine, the ipilimumab/dacarbazine combination resulted in an increased response rate (15% versus 5%) and improved one-, two-, and three-year survival [40]. In a single arm phase 2 study, the combination of ipilimumab and temozolamide resulted in an overall disease control rate (CR/PR and SD) of 67% which is much higher than seen in single agent studies [41]. A phase 1 study testing the combination of ipilimumab and bevacizumab resulted in a RR of 36% and overall disease control rate of 67%, but immune-related adverse reactions also seemed to be greatly enhanced with this combination [42]. Ipilimumab is also being combined with multiple other agents including granulocyte-macrophage colony-stimulating factor (GM-CSF), vaccines and other immune modulators with a goal to overcoming the immune tolerance of melanoma. In summary, there is no agent that is proven to increase the response rate of ipilimumab in a phase 3 trial and until that evidence exists, we do not recommend combining ipilimumab with any other therapy outside the setting of a clinical trial.

### Ipilimumab combined with radiation

Finally, an additional area of research to improve on the success of ipilimumab is through the combination of ipilimumab with radiation therapy with two trials currently enrolling patients. The first is a pilot study of ipilimumab in stage IV melanoma patients who are receiving palliative radiation therapy [43]. The second is the RADVAX study, a stratified phase 1/2 dose escalation trial of stereotactic body radiotherapy followed by ipilimumab in patients with metastatic melanoma [43]. The toxicity profile of this combination therapy will be of interest as both therapies can elicit similar toxicities such as colitis [44]. Trials combining vemurafenib and radiation therapy are also in the planning phases.

### Additional targeted therapies

As mentioned above, approximately 50% of patients with melanoma harbor BRAF mutations and are eligible for treatment with the novel BRAF inhibitors. In addition to BRAF, other mutations in genes as well as alterations in cancer related pathways have been identified in patients with melanoma, thereby leading investigators to target these pathways as well.

#### C-KIT (ED Query: I wasn't sure if this should be in all caps or all lower case so I left it as the authors had it.)

c-kit, also known as CD117, is a receptor tyrosine kinase that is mutated in approximately 20% of acral, mucosal, and chronically sun-damaged skin [45]. The ligand for KIT is stem cell factor (SCF) and binding of SCF to c-kit induces activation of downstream signaling pathways that are involved in mediating growth and survival signals within the cell including the P13K-AKT-mTOR pathway and the RAS-RAF-MEK-ERK pathway. KIT has been implicated in the pathogenesis of several cancers including acute myeloid leukemia and gastrointestinal stromal tumors (GIST) [46-49]. Unlike in GIST where c-kit mutations tend to be deletions or insertions in exon 11, c-kit mutations in melanoma occur at multiple sites along the gene including both the juxta-membrane domain at exon 11 and exon 13 and the kinase domain at exon 17 and are usually point mutations that do not correlate with KIT copy number or CD117 expression [50,51].

Imatinib, an oral tyrosine kinase inhibitor with known activity against c-kit activated tumors, was tested in three phase 2 studies of patients with melanomas that harbor c-kit mutations [52-54]. The first study enrolled patients with metastatic melanoma who expressed at least one protein tyrosine kinase (c-kit, platelet derived growth factor receptors, c-abl, or abl related gene) demonstrating a response in only one patient, interestingly in the patient who had the highest level of c-kit expression [52]. Of note, mutations in c-kit were not required prior to entry in this study. In the second study, 28 patients with c-kit mutations and amplifications with advanced unresectable melanoma arising from acral, mucosal, and chronically sun-damaged sites were treated with imatinib mesylate 400 mg orally twice daily in six-week cycles until disease progression or unacceptable toxicity [54]. Durable overall response rate (ORR) was 16% with a median time to progression of 12 weeks and median OS of 46.3 weeks. The third study enrolled 43 patients with c-kit mutated metastatic melanoma treated with imatinib who demonstrated an ORR of 23.3% and median OS of 14 months, with 51% alive at one year [53]. Please see Table 3 for a summary of these three trials. In summary, the responses to imatinib were primarily demonstrated in patients harboring mutations in c-kit at exon 11 or exon 13. Activity of dasatinib in c-kit mutated melanoma has already been demonstrated and additional trials with imatinib, nilotinib, and dasatinib are currently ongoing [43,50,55]. As is the case with the BRAF inhibitors, combinations of c-kit inhibitors with cyotoxic agents, immunotherapies, and other targeted therapies are underway.

**Table 3:** Summary of C-KIT Trials

Trial	Patients	RR% (CR/PR)	PFS (month)	OS	Number (patients)	Summary
Single Arm Phase 2 Kim KB *et al. *[52]	Imatinib mesylate 400 mg bid in advanced unresectable melanoma	5%	1.4	7.5 months	21	Imatinib mesylate demonstrated a response in 1 patient who also had high c-kit expression and alternate splicing variant in c-kit mRNA transcript.
Single Arm Phase 2 Carvajal *et al. *[54]	Imatinib mesylate 400 mg bid in advanced unresectable melanoma	16%	3	46.3 weeks	28	Imatinib mesylate demonstrated a significant clinical response in subset of patients with cKit mutation and advanced melanoma^a^
Single Arm Phase 2 Guo *et al. *[53]	Imatinib mesylate 400 mg daily in metastatic melanoma	23.3%	3.5	14 months	43	Imatinib mesylate demonstrated a significant clinical response in a subset of patients with cKit mutation and metastatic melanoma^a^

**^a^**the clustering of responses in both trials were seen in patients harboring c-kit mutations at exon 11 or exon 13; c-kit overexpression on IHC without a mutation did not correlate with a response. CR, complete response; OS, overall survival; PFS, progression free survival; PR, partial response; RR, response rate.

### ERBB4

ERBB4 (HER4) is a protein tyrosine kinase that activates both the ERK and AKT signaling pathways [56,57]. In a genome wide search of the tyrosine kinome, ERBB4 mutations were identified in 19% of patients with melanoma. *In vitro *assays demonstrated that lapatinib, a pan-ERBB inhibitor had activity in cell lines with these mutations and not in cell lines without these mutations [58]. In this same study, it was noted the ERBB4 mutations were found in patients with and without BRAF mutations suggesting that ERBB4 is perhaps an independent and complementary driver of tumorigenesis. A phase 2 trial of lapatanib in stage IV melanoma patients who harbor an ERBB4 mutation is currently enrolling [43]. Future therapeutic combinations of lapatinib and other ERBB4 inhibitors with either other targeted, immune, or chemotherapeutic agents hold promise.

### VEGF

Vascular endothelial growth factor (VEGF) as a mediator of tumor associated angiogenesis plays an essential role in the progression of melanoma [59]. The vascular radial and vertical growth patterns of melanoma are associated with rapid progression and metastasis, indicative of a therapeutic role for VEGF inhibition [60]. As single agents, anti-VEGF therapies have not demonstrated much success in patients with metastatic melanoma, but when combined with chemotherapy and immunotherapy there is some hint that anti-VEGF therapy has a future in the treatment of patients with metastatic melanoma. In a single arm phase 2 study of carboplatin, paclitaxel, and bevacizumab in patients with stage IV melanoma the RR was 17% with a median progression free survival (PFS) of six months and median OS of 12 months [61]; however, a randomized phase 2 trial of carboplatin and paclitaxel plus or minus bevacizumab failed to demonstrated significant improvement in OS (12.3 versus 8.6, *P *= 0.17) [62]. A single arm phase 2 study in chemotherapy naïve (CN) patients and previously treated (PT) patients tested the combination of bevacizumab with nab-paclitaxel and carboplatin and demonstrated a RR of 25.6% (CN) and 8.8% (PT) with median PFS of 4.5 (CN) months and 4.1 (PT) months and OS of 11.1 (CN) months and 10.1 (PT) months [63]. This combination is moving forward for a phase 3 evaluation. A multi-center phase 2 study of axitinib, an oral second-generation inhibitor of VEGF receptor-1, 2, and 3 demonstrated a RR of 19% with a median PFS of 3.9 months and median OS of 6.6 months [64]. In addition, a separate VEGF receptor-1, 2, and 3 inhibitor, E7080 or lenvatinib, demonstrated a RR of 21% in phase 1 evaluation of patients with solid tumors including patients with melanoma [65]. Taken together, inhibiting the VEGF pathway is likely beneficial for a subset of patients and improved patient selection combined will hopefully lead to clinically meaningful improvements in patient outcomes. Please see Table 4 summarizing the above trials involving VEGF in melanoma.

**Table 4:** Summary of VEGF Trials

Trial	Patients	RR% (CR/PR)	PFS (months)	OS (months)	Number (patients)	Summary
Single Arm Phase 2 Perez *et al. *[61]	Carboplatin plus paclitaxel and bevacizumab in unresectable metastatic melanoma	17	6	12	53	Carboplatin plus paclitaxel and bevacizumab was well tolerated and clinically beneficial
Randomized Phase 2 Kim *et al. *[62]	Carboplatin plus paclitaxel and bevacizumab versus Carboplatin plus paclitaxel in untreated metastatic melanoma	25.5 versus 16.4	5.6 versus 4.2	12.3 versus 8.6	214	Carboplatin plus paclitaxel and bevacizumab demonstrated statistically significant improvement in OS
Single Arm Phase 2 Kottschade *et al. *[63]	Carboplatin plus nab-paclitaxel in chemotherapy naïve(CN) and previously treated (PT) metastatic melanoma	25.6 (CN) 8.8 (PT)	4.5 (CN) 4.1 (PT)	11.1 (CN) 10.1 (PT)	41 (CN) 35 (PT)	Carboplatin plus nab-paclitaxel has clinical activity in chemotherapy naïve patients
Single Arm Phase 2 Fruehauf *et al. *[64]	Axitinib^a ^in metastatic melanoma after maximum on one prior therapy	18.8%	3.9	6.6	32	Axitinib demonstrated clinical activity in metastatic melanoma.

**^a^**Axitinib is an oral second-generation inhibitor of VEGF receptors 1, 2, and 3. CR, complete response; OS, overall survival; PFS, progression free survival; PR, partial response RR, response rate.

### Future of immunotherapy

#### PD-1/PD-L1

One mechanism by which melanoma is thought to evade the immune system is through tumor expression of programmed death ligand 1 (PD-L1). PD-L1 is a negative regulator of the immune system that acts through binding of the PD-1 present on activated lymphocytes and PD-L1/PD-1 interaction causes immune tolerance through apoptosis of the activated lymphocyte [66-68]. MDX-1106 is a genetically engineered fully human immunoglobulin G4 monoclonal antibody specific for human PD-1 [69]. A phase 1 study of anti-PD-1 antibody, MDX-1106, demonstrated single agent responses in a variety of previously treated, refractory solid tumors including melanoma with few treatment-related immune toxicities [69]. The clinical experience with anti-PD1 treatment is limited but encouraging and a dose escalation study of the combination of MDX-1106 and ipilimumab is currently recruiting [43]. In addition to antibodies that target PD-1, phase 1 trials of anti-PD-L1 are also underway.

#### Adoptive T cell therapy

The presence of tumor infiltrating lymphocytes (TIL) in resected melanoma samples is one of the reasons melanoma is often characterized as an immunogenic malignancy. Attempts to isolate, expand and infuse TIL for the treatment of cancer is termed adoptive cell therapy (ACT) and has shown promise for the treatment of patients with metastatic melanoma [70-73]. Early studies using TIL and IL-2 produced a response rate of 34%. Later studies using nonmyeloblative lymphodepletion with cytotoxic chemotherapy, with or without total body irradiation, induced response rates as high as 72% [70-75]. One of the limitations of ACT is that TIL expansion is not possible for all patients although it was recently reported that TIL is successfully generated in > 60% of all patients with melanoma and this figure is even higher in patients who did not receive prior chemotherapy [76]. In order to circumvent the process of tumor resection and isolation of TIL, investigators have developed methods to genetically modify autologous peripheral T cells to express a T cell receptor that targets melanoma antigens. This approach has induced a response rate in a subset of patients with melanoma and a similar approach has also yielded results in patients with refractory chronic lymphocytic leukemia [77]. In summary, ACT for the treatment of melanoma is a promising, albeit resource consuming method for the treatment of metastatic melanoma.

#### Interleukin-2

With the approval of ipilimumab, the role of HD IL-2 remains in question. While the clinical benefit of HD IL-2 is roughly similar to ipilimumab, the toxicity is worse and tolerability is less. Multiple strategies have attempted to identify biomarkers to predict clinical benefit of HD IL-2. Recently, a retrospective study found that patients with an NRAS mutation have a higher likelihood to respond to HD IL-2 [78]. In addition, a prospective study of patients with metastatic melanoma and renal cell carcinoma found that elevated pre-treatment serum VEGF and fibronectin are inversely correlated with the response rate to HD-IL-2 [79]. Both of these findings require further study at additional centers before changing clinical practice. Multiple efforts to increase the response rate of HD IL-2 while maintaining the duration of response have been attempted. The addition of a peptide vaccine (gp-100) to HD IL-2 demonstrated an improved objective response rate (16% versus 6%, *P *= 0.03) and overall survival (17.8 versus 11.1 months, *P *= 0.06) [80]. There are plans to improve upon this finding by using more potent vaccines.

Finally, it is becoming more evident that patients who progress on HD IL-2 do derive clinical benefit from ipilimumab. In the phase 3 study of previously treated patients, patients who progressed on prior HD-IL-2 received a similar benefit to ipilimumab as those who did not [24]. Similarly, a retrospective study analyzing patients who progressed on HD IL-2 and then received ipilimumab had a response rate (19%) and OS (12 months) to ipilimumab that was similar to previously reported historical controls [81]. Given the lack of approved agents and the limited but quantifiable benefit, we believe there still remains a role for HD IL-2 in patients who are fit enough to receive it.

### Combined immune and targeted therapy

As we learn more about the molecular pathways and immune modulation of melanoma, novel combinations of immune and targeted therapies have the potential of overcoming the low response rates of immune therapy and the short durations of response in targeted therapies. Pre-clinical work has suggested that BRAF inhibition leads to increased tumor recognition by T-cells providing a rational for the combination of BRAF inhibitors with agents that stimulate the immune system such as ipilimumab [82]. In addition, BRAF inhibitors and other targeted therapies will likely be combined with ipilimumab, IL-2, anti-PD-1, and other immunotherapies that are currently being tested.

### Role of surgery in metastatic disease

Patients with oligometastatic disease present a clinical dilemma of whether to treat systemically or locally. Highly selected patients with isolated lung or liver metastasis, good performance status, and less aggressive tumor biology have benefited from metastasectomy with improved five-year survival rates and median OS rates of 20 to 25 months [83-87]. Again, patient selection is the key for success in this setting. A recent phase 2 study enrolled 77 patients for complete resection of metastatic disease and demonstrated a relapsed free survival of five months and 36% of patients alive at three years [88]. While this trial demonstrates that long-term survival can be achieved through surgery, the trial did not further identify patients more likely to benefit from surgery. Currently, a prospective trial of patients with oligometastatic disease is randomizing patients to either surgery versus systemic therapy. This will hopefully shed more light on which patients are most likely to benefit from metastasectomy.

## Conclusions

In 2011, the FDA approval of vermurafenib and ipilimumab instilled optimism in clinicians treating patients with metastatic melanoma. While these therapies have limitations, many promising strategies exist to overcome these limitations. Understanding and overcoming resistance pathways, combining current and future agents, identifying biomarkers to improve patient selection, and discovering future therapeutic targets will hopefully lead to further treatment advances.

## Abbreviations

ACT: Adoptive cell therapy; CN: chemotherapy naïve; CR: complete response; CTLA4: cytotoxic T-lymphocyte-associated antigen 4; GIST: gastrointestinal stromal tumors; Gp100: glycoprotein 100 peptide vaccine; GM-CSF: granulocyte-macrophage colony-stimulating factor; HD IL-2: high dose IL-2; ICOS: inducible co-stimulatory molecule; MAP: mitogen activated kinase pathway; ORR: overall response rate; OS: overall survival; PR: partial response; PT: previously treated; PD-L1: programmed death ligand 1; PFS: progression free survival; RR: response rate; SCC: squamous cell carcinoma; SD: stable disease; SCF: stem cell factor; TIL: tumor infiltrating lymphocytes; VEGF: vascular endothelial growth factor.

## Competing interests

The authors declare that they have no competing interests.

## Authors' contributions

LF wrote the initial version of the manuscript. SM critically reviewed the manuscript. RJ conceived the outline of the manuscript, reviewed the manuscript, and made final changes. All authors read and approved the final manuscript.

## Authors' information

LF is a fellow in Hematology and Oncology at Mayo Clinic Florida. SM is a Professor of Medicine and Oncology at Mayo Clinic Rochester with a clinical and research focus on melanoma. RJ is a Clinical Instructor at Mayo Clinic Florida with a clinical and research focus on melanoma and genitourinary malignancies.

## Pre-publication history

The pre-publication history for this paper can be accessed here:

http://www.biomedcentral.com/1741-7015/10/23/prepub
